# Mayo Clinic Electrophysiology Manual. Editors: Sameul J Asirvatham, Yong-Mei Cha, Paul A Friedman. Mayo Clinic Scientific Press

**Published:** 2014-05-25

**Authors:** Johnson Francis

**Affiliations:** Senior Consultant Interventional Cardiologist, Baby Memorial Hospital, Calicut, Kerala, India

*Mayo Clinic Electrophysiology Manual* is a concise book on cardiac electrophysiology from the Mayo Clinic Scientific Press, published by the Oxford University Press. Preface from the Editor-in-Chief Prof. Samuel J Asirvatham ends with a very interesting note: "If there is one regret in choosing a career in cardiac electrophysiology, it is that I can never again experience the unparalleled wonder and joy of discovering the nuances and hidden treasures of learning electrophysiology for the first time". It is this very joy that the book delivers to a first time reader as well!

The introductory chapter runs into 64 pages and details the fluoroscopic views used in cardiac electrophysiology, amply illustrated with corresponding intra cardiac electrograms. Pictures of anatomic dissections, drawings, three dimensional tomographic reconstructions as well as intracardiac ultrasound images are provided for better correlation and understanding, where ever needed. Clarity of drawings is superb, like in all other publications from Mayo Clinic, with support from an excellent medical illustration wing.

Second chapter dedicated to intracardiac echocardiography is also well illustrated with anatomic sections and drawings. Third chapter on electroanatomic mapping details all the various contact and non contact three dimensional mapping technologies currently available. This chapter adds a note on ultrasound based catheter navigation and remote magnetic navigation systems. Remote magnetic navigations systems have the potential to reduce radiation to the patient and virtually eliminating the radiation exposure to the operator.

Fourth chapter is on various diagnostic maneuvers in the EP lab. Various pacing protocols are discussed in detail. Fifth chapter delves into the mechanisms of reentry and entrainment while the sixth is an approach to wide QRS tachycardia. Basic electrophysiology at the cellular level, ion channels and channelopathies comprise the next chapter. Anti arrhythmic drugs as relevant to the ablationist is dealt with in eighth chapter while the next one deals with catheter ablation and device therapy in congenital heart disease.

Rest of the book is devoted to illustrative case presentations. Twenty cases with plenty of elctrograms, fluoroscopic views and anatomical drawings and sections make the second section of the book very interesting and informative. Overall, this EP manual is one of the best for the electrophysiologist in training and good refresher for the practicing consultant.

